# Polymorphisms within the Novel Type 2 Diabetes Risk Locus *MTNR1B* Determine β-Cell Function

**DOI:** 10.1371/journal.pone.0003962

**Published:** 2008-12-17

**Authors:** Harald Staiger, Fausto Machicao, Silke A. Schäfer, Kerstin Kirchhoff, Konstantinos Kantartzis, Martina Guthoff, Günther Silbernagel, Norbert Stefan, Hans-Ulrich Häring, Andreas Fritsche

**Affiliations:** Division of Endocrinology, Diabetology, Angiology, Nephrology, and Clinical Chemistry, Department of Internal Medicine, University Hospital Tübingen, Tübingen, Germany; University of Bremen, Germany

## Abstract

**Background:**

Very recently, a novel type 2 diabetes risk gene, i.e., *MTNR1B*, was identified and reported to affect fasting glycemia. Using our thoroughly phenotyped cohort of subjects at an increased risk for type 2 diabetes, we assessed the association of common genetic variation within the *MTNR1B* locus with obesity and prediabetes traits, namely impaired insulin secretion and insulin resistance.

**Methodology/Principal Findings:**

We genotyped 1,578 non-diabetic subjects, metabolically characterized by oral glucose tolerance test, for five tagging single nucleotide polymorphisms (SNPs) covering 100% of common genetic variation (minor allele frequency >0.05) within the *MTNR1B* locus (rs10830962, rs4753426, rs12804291, rs10830963, rs3781638). In a subgroup (N = 513), insulin sensitivity was assessed by hyperinsulinemic-euglycemic clamp, and in a further subgroup (N = 301), glucose-stimulated insulin secretion was determined by intravenous glucose tolerance test. After appropriate adjustment for confounding variables and Bonferroni correction for multiple comparisons, none of the tagging SNPs was reliably associated with measures of adiposity. SNPs rs10830962, rs4753426, and rs10830963 were significantly associated with higher fasting plasma glucose concentrations (p<0.0001) and reduced OGTT- and IVGTT-induced insulin release (p≤0.0007 and p≤0.01, respectively). By contrast, SNP rs3781638 displayed significant association with lower fasting plasma glucose levels and increased OGTT-induced insulin release (p<0.0001 and p≤0.0002, respectively). Moreover, SNP rs3781638 revealed significant association with elevated fasting- and OGTT-derived insulin sensitivity (p≤0.0021). None of the *MTNR1B* tagging SNPs altered proinsulin-to-insulin conversion.

**Conclusions/Significance:**

In conclusion, common genetic variation within *MTNR1B* determines glucose-stimulated insulin secretion and plasma glucose concentrations. Their impact on β-cell function might represent the prevailing pathomechanism how *MTNR1B* variants increase the type 2 diabetes risk.

## Introduction

During the last two years, genome-wide association (GWA) studies based on tens of thousands of human cases and controls identified a series of novel type 2 diabetes risk loci including *SLC30A8*, *HHEX*, *CDKAL1*, *IGF2BP2*, and *CDKN2A/B* [1]–[5] which were subsequently replicated in other human cohorts and ethnicities [6]–[13]. Further investigations in thoroughly phenotyped cohorts revealed that these gene variants or, more precisely, these single nucleotide polymorphisms (SNPs) affect insulin secretion, but not insulin sensitivity [9;14]–[20].

Very recently, an additional type 2 diabetes risk gene, i.e., *MTNR1B*, was identified and reported to affect fasting plasma glucose concentrations [21]; [22]. *MTNR1B* (OMIM entry #600804) is located on human chromosome 11q21–q22 and encodes one of the two high-affinity G-protein-coupled receptors for the pineal gland hormone melatonin. Due to its predominant expression in retina and brain [23], melatonin receptor 1B is thought to participate in light-dependent functions in the retina and in melatonin's neuronal regulation of circadian rhythmicity and sleep cycles. As certain sleep disorders, such as obstructive sleep apnea, result from obesity and are associated with insulin resistance [24]; [25], *MTNR1B* could represent a new interesting candidate gene linking sleep disorders with type 2 diabetes.

Since *MTNR1B*'s role in the pathogenesis of human obesity and human prediabetic phenotypes, such as insulin resistance and β-cell dysfunction, was not yet assessed, it was the aim of the present study to analyse, using a HapMap approach, the association of common genetic variation (minor allele frequency, MAF>0.05) within the *MTNR1B* locus with state-of-the art measures of obesity, glucose tolerance, insulin sensitivity, and β-cell function in a thoroughly phenotyped population at an increased risk for type 2 diabetes.

## Methods

### Subjects

One thousand six hundred and seventy-five subjects were recruited from the ongoing Tübingen Family Study for type 2 diabetes (TÜF). The publicly announced call for TÜF primarily addressed non-diabetic individuals from Southern Germany with family history of type 2 diabetes or diagnosis of impaired fasting glycemia. At least 99.5% of the TÜF participants are of European ancestry. Selection of the present study cohort was based on the availability of DNA samples, proinsulin and C-peptide measurements, and complete datasets. From the 1,675 subjects, 97 were excluded due to newly diagnosed type 2 diabetes. This exclusion resulted in a non-diabetic cohort of 1,578 subjects (for glucose tolerance status, see Table 1). 68% of these subjects had a recorded family history of type 2 diabetes. All participants underwent the standard procedures of the protocol including medical history and physical examination, assessment of smoking status and alcohol consumption habits, routine blood tests, and an oral glucose tolerance test (OGTT). A subgroup of 513 subjects agreed to undergo a hyperinsulinemic-euglycemic clamp. Another subgroup of the clamped subjects (N = 301) additionally agreed to undergo an intravenous glucose tolerance test (IVGTT). The participants were not taking any medication known to affect glucose tolerance or insulin secretion. Informed written consent to the study was obtained from the participants, and the local ethics committee (Ethik-Kommission der Medizinischen Fakultät der Universität Tübingen) approved the study protocol.

**Table 1:** Clinical characteristics of the study population.

Gender (female/male)	1044/534
NGT/IFG/IGT/(IFG+IGT)	1139/164/152/123
Age (y)	40±13
BMI (kg/m^2^)	28.9±8.2
Waist circumference (cm)	94±17
Fasting glucose (mM)	5.11±0.55
Glucose, 120 min OGTT (mM)	6.27±1.66
Fasting insulin (pM)	63.7±52.9
Insulin, 30 min OGTT (pM)	493±393

Data are given as means±SD. BMI–body mass index; IFG–impaired fasting glucose; IGT–impaired glucose tolerance; NGT–normal glucose tolerance; OGTT–oral glucose tolerance test.

### Selection of tagging SNPs and genotyping

**Figure 1:**
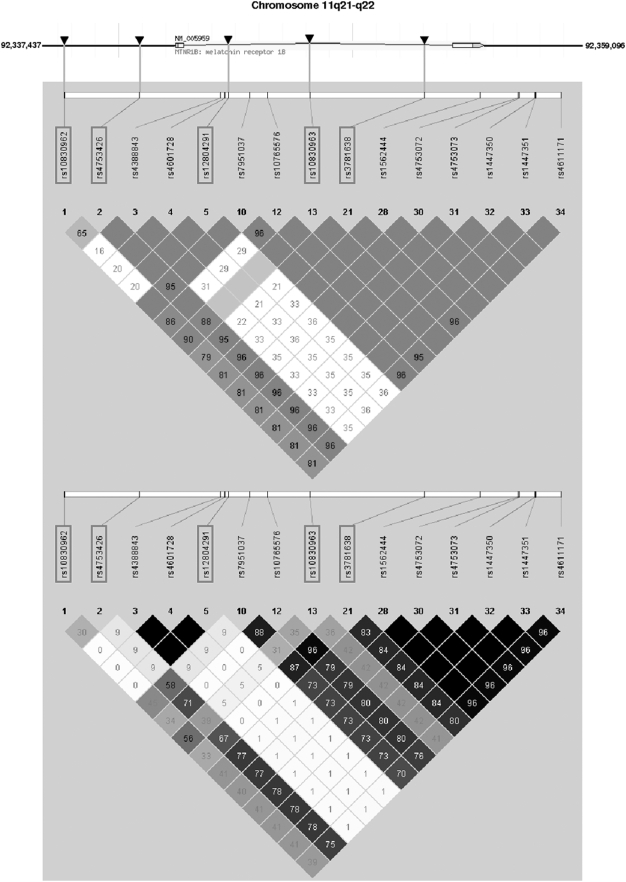
Genomic region of human chromosome 11q21–q22 harbouring the *MTNR1B* gene and HapMap linkage disequilibrium data of common (MAF>0.05) informative SNPs within this region. The *MTNR1B* gene consists of two exons and one intron and spans 13.16 kb from nucleotide 92,342,437 to nucleotide 92,355,596. The analysed region additionally included 5 kb of the 5′-flanking region and 3.5 kb of the 3′-flanking region. The locations of the five tagging SNPs (highlighted by red frames) are indicated by arrows. In the upper panel, D' values are given in the diamonds (red diamonds without values: D' = 1.0). In the lower panel, r^2^ values are given in the diamonds (black diamonds: r^2^ = 1.0).

Using the publically available phase II data of the International HapMap Project derived from a population of Utah residents with ancestry from Northern and Western Europe (release #23a March 2008, http://www.hapmap.org/index.html.en, [26]), we screened in silico the complete *MTNR1B* gene spanning 13.16 kb (two exons, one intron, located on human chromosome 11q21–q22) as well as 5 kb and 3.5 kb of its 5′- and 3′-flanking regions, respectively (Figure 1). Within this locus, 15 informative SNPs with MAF>0.05 were present, and their HapMap linkage disequilibrium data (D' and r^2^ values) are given in Figure 1. Among these, five tagging SNPs were selected covering 100% of the common genetic variation (MAF>0.05) within this locus with an r^2^>0.8 based on Tagger analysis using Haploview software (http://www.broad.mit.edu/mpg/haploview, [27]). The five tagging SNPs rs10830962 C/G and rs4753426 T/C (both located in the 5′-flanking region of the gene, Figure 1), rs12804291 C/T, rs10830963 C/G, and rs3781638 A/C (all three located within the single intron, Figure 1) were selected for genotyping. For genotyping, DNA was isolated from whole blood using a commercial DNA isolation kit (NucleoSpin, Macherey & Nagel, Düren, Germany). SNPs were genotyped using TaqMan assays (Applied Biosystems, Foster City, CA, USA). The TaqMan genotyping reaction was amplified on a GeneAmp PCR system 7000 (50°C for 2 min, 95°C for 10 min, followed by 40 cycles of 95°C for 15 s and 60°C for 1 min), and fluorescence was detected on an ABI Prism sequence detector (Applied Biosystems, Foster City, CA, USA). The TaqMan assays were validated by direct sequencing of the SNPs in 50 subjects, and both methods gave identical results. The overall genotyping success rate was 99.9% (rs10830962: 99.9%, rs4753426: 99.9%, rs12804291: 100%, rs10830963: 100%, and rs3781638: 99.8%), and rescreening of 3.3% of the subjects with the TaqMan assay gave 100 % identical results.

### Determination of adiposity

Percentage of body fat was measured using bioelectrical impedance (BIA-101, RJL systems, Detroit, MI, USA). Body mass index (BMI) was calculated as weight divided by squared height. Waist circumference was measured in the upright position at the midpoint between the lateral iliac crest and the lowest rib.

### Determination of insulin secretion and insulin sensitivity

After a 10-h overnight fast, all subjects underwent a 75-g OGTT, and venous blood samples were obtained at 0, 30, 60, 90, and 120 min for determination of plasma glucose, insulin, proinsulin, and C-peptide. In those subjects who agreed to undergo both the IVGTT and the hyperinsulinemic-euglycemic clamp, the IVGTT was performed prior to the clamp after a 10-h overnight fast, as described by the Botnia protocol [28]. After baseline samples (−10 and −5 min) had been collected, a glucose dose of 0.3 g/kg body weight was given at time 0. Blood samples for the measurement of plasma glucose, insulin, and C-peptide were obtained at 2, 4, 6, 8, 10, 20, 30, 40, 50, and 60 min. The hyperinsulinemic-euglycemic clamp was performed starting at 60 min after the IVGTT glucose bolus. To this end, subjects received a primed infusion of short-acting human insulin (40 mU/m^2^/min) for 120 min. Variable infusion of 20% glucose was started to clamp the plasma glucose concentration at fasting levels. Blood samples for the measurement of plasma glucose were obtained at 5-min intervals. Plasma insulin levels were measured at baseline (prior to the IVGTT glucose bolus) and at the steady state (the last 30 min) of the clamp. In subjects who agreed to undergo the hyperinsulinemic-euglycemic clamp, but not the IVGTT, the clamp was started after the 10-h overnight fast.

### Determination of blood parameters

Plasma glucose was determined using a bedside glucose analyzer (glucose oxidase method, Yellow Springs Instruments, Yellow Springs, CO, USA). Plasma insulin and C-peptide concentrations were measured by commercial chemiluminescence assays for ADVIA Centaur (Siemens Medical Solutions, Fernwald, Germany) according to the manufacturer's instructions. Plasma proinsulin was determined by microparticle enzyme immunoassay (IBL, Hamburg, Germany). The proinsulin assay has 0% cross-reactivity with human insulin and C-peptide, the insulin assay has 0% cross-reactivity with proinsulin.

### Calculations

The area under the curve (AUC) of plasma glucose levels during the OGTT was calculated according to the trapezoid method as: 0.5[0.5c(glucose)_0_+c(glucose)_30_+c(glucose)_60_+c(glucose)_90_+0.5c(glucose)_120_]. The AUC of plasma insulin, C-peptide, and proinsulin levels during the OGTT was calculated analogously. Insulin secretion during the OGTT was assessed by calculating the ratio AUC C-peptide divided by AUC glucose (AUC C-pep/AUC glc), first-phase insulin secretion, and the insulinogenic index. First-phase insulin secretion was estimated from plasma insulin and glucose concentrations during the OGTT using the formerly described equation [29]: 1,283+1.829c(insulin)_30_−138.7c(glucose)_30_+3.772c(insulin)_0_. The insulinogenic index was calculated as: [c(insulin)_30_−c(insulin)_0_]/[c(glucose)_30_−c(glucose)_0_]. Homeostasis model assessment of insulin resistance (HOMA-IR) was calculated as [c(glucose)_0_·c(insulin)_0_]/22.5. Insulin sensitivity from OGTT was estimated as proposed by Matsuda and DeFronzo [30]: 10,000/[c(glucose)_0_·c(insulin)_0_·c(glucose)_mean_·c(insulin)_mean_]^½^. Clamp-derived insulin sensitivity was calculated as glucose infusion rate necessary to maintain euglycemia during the last 40 min (steady state) of the clamp divided by the steady-state insulin concentration. The disposition index was calculated as first-phase insulin secretion multiplied by OGTT-derived insulin sensitivity. Proinsulin conversion was estimated as fasting proinsulin divided by fasting insulin and, during the OGTT, as AUC proinsulin divided by AUC insulin. Estimates of hepatic insulin clearance were obtained during the OGTT by dividing AUC C-peptide by AUC insulin and mean C-peptide by mean insulin.

### Statistical analyses

Hardy-Weinberg equilibrium was tested using χ^2^ test. Linkage disequilibrium between the tagging SNPs was analysed using the JLIN program provided by the Western Australian Institute for Medical Research (http://www.genepi.org.au/jlin [31]). Prior to regression analysis, all continuous data were log-transformed in order to approximate normal distribution. To adjust for confounding variables, multivariate linear regression models were applied, and the trait of interest (e.g., BMI, insulin sensitivity index, or insulin secretion index) was chosen as dependent variable. Multivariate linear regression analysis was performed using the least-squares method. Differences in C-peptide levels during the IVGTT were tested using repeated-measures multivariate analysis of variance (MANOVA). Based on testing five non-linked SNPs and three independent parameters, i.e., measures of adiposity, measures of insulin secretion, and measures of insulin action, we performed 15 independent statistical tests in the OGTT group. Therefore, a p-value <0.0034 was considered statistically significant in this group according to Bonferroni correction for multiple comparisons. In the clamp and IVGTT subgroups, we tested the five tagging SNPs and only one parameter, i.e., insulin sensitivity or C-peptide levels, respectively, and therefore performed five independent statistical tests. Accordingly, a p-value <0.0102 was considered statistically significant in these subgroups. To perform these analyses, the statistical software package JMP 4.0 (SAS Institute, Cary, NC, USA) was used. Using F-test (one-way ANOVA with fixed effects), our OGTT study (N = 1578) was sufficiently powered (1-β>0.8, α<0.0034) to detect effect sizes as small as 10% (additive inheritance model). In the clamp subgroup (N = 513), the study was sufficiently powered to detect effect sizes as small as 16% (1-β>0.8, α<0.0102; additive inheritance model), and in the IVGTT subgroup (N = 301), we were able to detect effect sizes as small as 20% (1-β>0.8, α<0.0102; dominant inheritance model). Power calculations were performed using G*power 3.0 software available at http://www.psycho.uni-duesseldorf.de/aap/projects/gpower/.

## Results

### Genotyping

We genotyped 1,578 non-diabetic subjects at an increased risk for type 2 diabetes (clinical characteristics given in Table 1) for the five *MTNR1B* tagging SNPs rs10830962, rs4753426, rs12804291, rs10830963, and rs3781638. As expected for tagging SNPs, these SNPs were not in strong linkage disequilibrium (Table 2). All SNPs were in Hardy-Weinberg equilibrium (all p>0.7) and displayed MAFs very close to those reported by the HapMap project (http://www.hapmap.org/index.html.en, [26], Table 2).

**Table 2:** Linkage disequilibrium statistics (D', r^2^) among the five tagging SNPs rs10830962, rs4753426, rs12804291, rs10830963, and rs3781638 covering the 21.66-kb genomic locus harbouring the *MTNR1B* gene.

SNP	rs10830962	rs4753426	rs12804291	rs10830963	rs3781638
rs10830962	-	0.638	0.326	0.965	0.821
rs4753426	0.288	-	1.000	0.969	0.927
rs12804291	0.018	0.114	-	1.000	0.488
rs10830963	0.581	0.414	0.051	-	1.000
rs3781638	0.357	0.643	0.022	0.330	-
MAF (MAF_HapMap_)	0.407 (0.383)	0.493 (0.458)	0.106 (0.108)	0.300 (0.300)	0.435 (0.466)

D' values above empty cells; r^2^ values below empty cells. SNP–single nucleotide polymorphism, MAF–minor allele frequency

### Anthropometrics, OGTT- and clamp-derived data

These analyses were performed in the additive inheritance model. After adjustment for gender, age, and family history of diabetes and Bonferroni correction for multiple comparisons (corrected α-level: p<0.0034), none of the tagging SNPs was reliably associated with measures of adiposity, such as BMI, body fat content, and waist circumference (Tables 3 and 4). SNPs rs10830962, rs4753426, and rs10830963 revealed marked associations with higher fasting plasma glucose concentrations (all p<0.0001 after adjustment for gender, age, BMI, and family history of diabetes), with lower values in all OGTT-derived measures of insulin release (all p≤0.0007 after adjustment for gender, age, BMI, insulin sensitivity, and family history of diabetes; Tables 3 and 4), and with lower values of the disposition index (all p<0.0001 after adjustment for gender, age, BMI, and family history of diabetes). Interestingly, SNP rs3781638 displayed opposite effects (Table 4): this SNP was associated with reduced fasting plasma glucose concentrations (p<0.0001), with higher values in all OGTT-derived measures of insulin release (p≤0.0002), and with a higher value of the disposition index (p<0.0001). Moreover, SNP rs3781638 showed significant associations with increased OGTT-derived insulin sensitivity (p<0.0021 after adjustment for gender, age, BMI, and family history of diabetes) and correspondingly decreased HOMA-IR (p≤0.0015 after analogous adjustment; Table 4). We then tested whether this SNP also affects hepatic insulin clearance, a close correlate of liver insulin sensitivity, using the ratios AUC C-peptide/AUC insulin and mean C-peptide/mean insulin during the OGTT as estimates. After adjustment for gender, age, BMI, and family history of diabetes, SNP rs3781638 was significantly associated with these measures in the overall cohort as well as in the clamped subgroup (all p≤0.0199). None of the other tagging SNPs revealed associations with measures of insulin sensitivity (Tables 3 and 4). SNP rs12804291 was not associated with any of the traits tested (Table 3).

**Table 3:** Associations of *MTNR1B* SNPs rs10830962, rs4753426, and rs12804291 with anthropometrics, insulin sensitivity, and insulin secretion (N = 1578).

SNP	rs10830962					rs4753426					rs12804291				
Genotype	CC	CG	GG	p_1_	p_2_	TT	TC	CC	p_1_	p_2_	CC	CT	TT	p_1_	p_2_
N	548	783	246	-	-	407	780	391	-	-	1262	298	18	-	-
Age (y)	40±13	39±13	39±13	-	-	40±13	39±14	39±13	-	-	40±13	39±13	40±12	-	-
BMI (kg/m^2^)	29.2±8.4	28.5±8.1	29.2±8.2	0.2	0.1	29.3±8.2	28.5±8.1	29.1±8.4	0.2	0.2	28.8±8.2	28.8±8.1	31.6±11.8	0.5	0.4
Body fat (%)	32±11	31±11	31±11	0.09	0.08	32±10	31±11	31±11	0.0255	0.0252	31±11	31±11	35±10	0.2	0.2
Waist circum-ference (cm)	95±17	93±17	96±17	0.2	0.2	95±18	93±17	95±18	0.2	0.2	94±17	94±17	93±20	0.8	0.9
Fasting glucose (mM)	5.05±0.54	5.12±0.53	5.18±0.59	**<0.0001**	**<0.0001**	5.05±0.54	5.07±0.53	5.22±0.58	**<0.0001**	**<0.0001**	5.12±0.56	5.06±0.52	5.11±0.49	0.4	0.3
Glucose 120 min OGTT (mM)	6.15±1.65	6.33±1.66	6.34±1.65	0.0119	0.0153	6.26±1.67	6.20±1.63	6.42±1.69	0.07	0.06	6.26±1.66	6.29±1.67	6.13±1.34	0.7	0.7
HOMA-IR (U)	2.48±2.03	2.43±2.23	2.63±2.59	0.8	0.6	2.48±1.93	2.36±2.16	2.71±2.60	0.1	0.09	2.47±2.12	2.49±2.66	2.76±1.58	0.8	0.7
ISI, OGTT (U)	16.2±11.0	16.3±10.4	16.6±11.3	0.7	0.6	16.1±10.8	16.7±10.7	15.7±10.8	0.1	0.1	16.3±10.8	16.5±10.8	13.5±7.7	1.0	0.9
ISI, clamp (U)*	0.085±0.064	0.085±0.050	0.088±0.052	1.0	1.0	0.083±0.058	0.087±0.055	0.085±0.050	0.6	0.6	0.086±0.056	0.086±0.052	0.054±0.025	0.3	0.4
1^st^-phase insulin secretion (nM)	1.36±0.87	1.22±0.80	1.24±0.90	**<0.0001**	**<0.0001**	1.36±0.89	1.24±0.78	1.25±0.91	**<0.0001**	**<0.0001**	1.27±0.85	1.28±0.83	1.40±0.68	0.7	0.7
Insulinogenic index (·10^−9^)	174±258	129±406	128±177	**<0.0001**	**<0.0001**	126±538	161±244	131±161	**<0.0001**	**0.0001**	154±215	100±622	172±131	0.7	0.8
AUC C-pep/AUC glc (·10^−9^)	332±110	317±105	307±108	**0.0007**	**0.0007**	330±110	320±105	310±109	**0.0003**	**0.0006**	321±108	319±106	326±115	1.0	1.0
Disposition index (U)	17.1±9.0	15.6±8.3	15.5±9.3	**<0.0001**	**<0.0001**	16.8±8.9	16.4±8.5	14.7±8.7	**<0.0001**	**<0.0001**	16.1±8.8	16.4±8.5	15.1±4.9	0.7	0.6

Data represent means±SD. For statistical analysis, data were log-transformed and adjusted. BMI, body fat, and waist circumference were adjusted for gender and age. Plasma glucose levels, indices of insulin sensitivity, and the disposition index were adjusted for gender, age, and BMI. Other indices of insulin secretion were adjusted for gender, age, BMI, and ISI (OGTT). p_1_–p-value after adjustment as described; p_2_–p-value after additional adjustment for family history of diabetes. Significance levels withstanding Bonferroni correction for multiple comparisons are marked in bold letters. AUC–area under the curve; HOMA-IR–homeostasis model assessment of insulin resistance; ISI–insulin sensitivity index; SNP–single nucleotide polymorphism. ^*^subgroup (N = 513).

**Table 4:** Associations of *MTNR1B* SNPs rs10830963 and rs3781638 with anthropometrics, insulin sensitivity, and insulin secretion (N = 1578).

SNP	rs10830963					rs3781638				
Genotype	CC	CG	GG	p_1_	p_2_	AA	AC	CC	p_1_	p_2_
N	778	660	140	-	-	501	785	289	-	-
Age (y)	40±13	39±13	40±13	-	-	39±13	40±14	39±13	-	-
BMI (kg/m^2^)	29.0±8.2	28.6±8.1	29.0±8.6	0.6	0.3	29.0±8.6	28.7±8.1	29.0±8.0	0.8	0.7
Body fat (%)	31±11	31±11	30±12	0.4	0.3	31±11	31±11	31±10	0.2	0.2
Waist circum-ference (cm)	94±17	93±17	94±18	0.5	0.5	94±18	93±17	95±17	0.5	0.4
Fasting glucose (mM)	5.04±0.53	5.14±0.56	5.27±0.59	**<0.0001**	**<0.0001**	5.18±0.58	5.09±0.51	5.02±0.56	**<0.0001**	**<0.0001**
Glucose 120 min OGTT (mM)	6.20±1.65	6.32±1.64	6.41±1.75	0.08	0.07	6.35±1.66	6.26±1.64	6.14±1.69	0.09	0.09
HOMA-IR (U)	2.48±2.14	2.45±2.18	2.60±2.86	0.8	0.8	2.74±2.74	2.28±1.87	2.54±2.07	**0.0014**	**0.0015**
ISI, OGTT (U)	16.1±10.7	16.3±10.6	17.3±12.0	0.7	0.5	15.6±10.6	16.8±10.8	16.1±10.9	**0.0023**	**0.0021**
ISI, clamp (U)*	0.084±0.058	0.086±0.050	0.088±0.059	0.4	0.4	0.086±0.051	0.083±0.049	0.092±0.073	0.7	0.6
1^st^-phase insulin secretion (nM)	1.36±0.87	1.20±0.79	1.16±0.88	**<0.0001**	**<0.0001**	1.27±0.89	1.22±0.77	1.40±0.94	**<0.0001**	**<0.0001**
Insulinogenic index (·10^−9^)	154±443	141±158	105±193	**<0.0001**	**<0.0001**	136±156	151±286	141±584	**<0.0001**	**<0.0001**
AUC C-pep/AUC glc (·10^−9^)	330±109	313±104	299±109	**<0.0001**	**<0.0001**	315±110	316±103	340±112	**0.0001**	**0.0002**
Disposition index (U)	17.0±8.8	15.3±8.3	15.0±9.7	**<0.0001**	**<0.0001**	15.1±8.5	16.4±8.4	17.4±9.7	**<0.0001**	**<0.0001**

Data represent means±SD. For statistical analysis, data were log-transformed and adjusted. BMI, body fat, and waist circumference were adjusted for gender and age. Plasma glucose levels, indices of insulin sensitivity, and the disposition index were adjusted for gender, age, and BMI. Other indices of insulin secretion were adjusted for gender, age, BMI, and ISI (OGTT). p_1_–p-value after adjustment as described; p_2_–p-value after additional adjustment for family history of diabetes. Significance levels withstanding Bonferroni correction for multiple comparisons are marked in bold letters. AUC–area under the curve; HOMA-IR–homeostasis model assessment of insulin resistance; ISI–insulin sensitivity index; SNP–single nucleotide polymorphism. ^*^subgroup (N = 513).

After stratification of the cohort in normal glucose-tolerant subjects (N = 1139) and subjects with impaired fasting glycemia and/or impaired glucose tolerance (N = 439), all SNPs reported above to be significantly associated with fasting plasma glucose concentrations and measures of insulin secretion retained at least nominal associations with these traits in both subgroups (Supplementary Tables S1, S2, S3 and S4). Interestingly, the association of SNP rs3781638 with insulin sensitivity remained nominal in subjects with impaired fasting glycemia and/or impaired glucose tolerance, but was no longer seen in normal glucose-tolerant subjects (Supplementary Tables S2 and S4).

We recently reported that type 2 diabetes risk alleles in *TCF7L2*, *CDKAL1*, and *SLC30A8* impair proinsulin-to-insulin conversion [15]. Based on the same parameters, i.e., the ratio fasting proinsulin divided by fasting insulin and the ratio AUC proinsulin divided by AUC insulin, we could not detect any reliable association of the *MTNR1B* tagging SNPs with proinsulin conversion (all p≥0.04).

### IVGTT

**Figure 2:**
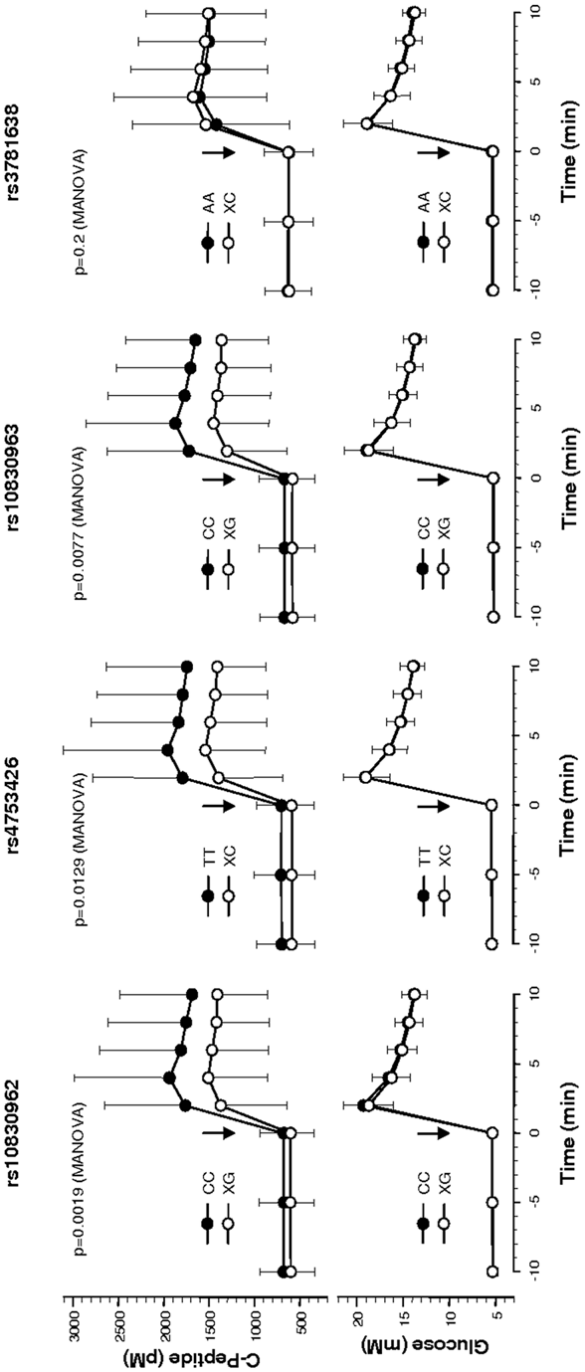
Plasma C-peptide and glucose concentrations during the IVGTT in carriers of the *MTNR1B* SNPs rs10830962, rs4753426, rs10830963, and rs3781638. Data are given as means±SD. The response of plasma C-peptide concentrations on time vs. genotype (dominant model) independent of gender, age, BMI, insulin sensitivity, and family history of diabetes was tested using repeated measures MANOVA. Arrows mark the time-point of glucose infusion. Black circles–homozygous carriers of the major allele; white circles: heterozygous and homozygous carriers of the minor allele.

In this study, we assessed whether the *MTNR1B* tagging SNPs affect glucose-stimulated insulin release. Due to the small sample size of the IVGTT subgroup, we performed these analyses in the dominant inheritance model. After adjustment for gender, age, BMI, insulin sensitivity, and family history of diabetes and Bonferroni correction for multiple comparisons (corrected α-level: p<0.0102), two of the four SNPs which were associated with OGTT-derived insulin secretion, namely rs10830962 and rs10830963, revealed significantly impaired glucose-stimulated insulin secretion (p = 0.0019 and p = 0.0077, respectively, Figure 2). The association of SNP rs4753426 with reduced C-peptide levels showed borderline significance (p = 0.0129, Figure 2), and SNP rs12804291 was not associated at all (p = 0.6).

## Discussion

With our non-diabetic cohort thoroughly phenotyped for prediabetic traits, we were recently able to gain important new information about the pathophysiological role of GWA-derived type 2 diabetes risk genes [14]; [15]; [32]; [33]. In the present study, we assessed the impact of common genetic variation within the novel type 2 diabetes risk gene *MTNR1B* on the pathogenesis of obesity and the prediabetic phenotypes insulin resistance and β-cell dysfunction.

The present study cohort encompassed a wide range of BMI (16.3–76.9 kg/m^2^), body fat content (7–67%), and waist circumference values (52–183 cm). Thus, the lack of association between common genetic variation within *MTNR1B* and these measures of body adiposity, as observed in this study, prompts us to suggest that this gene plays, if at all, only a minor part in the development of obesity. Nevertheless, further replication in larger studies is required to ultimately exclude a role of *MTNR1B* in this prediabetic trait.

By contrast, four of the five tagging SNPs revealed clear associations with insulin release upon an oral glucose load and three of them showed additional associations with glucose-stimulated insulin secretion in the IVGTT. Notably, the minor allele carriers of the latter SNPs, i.e., rs10830962, rs4753426, and rs10830963, revealed ∼20-% reductions in insulin secretion, as estimated from the IVGTT C-peptide data. Thus, these SNPs' marked effects on β-cell function are very likely to cause the alterations in fasting plasma glucose levels seen in this and earlier studies [21]; [22]. Furthermore, the association of SNP rs10830962 with 2-h plasma glucose levels might point to this gene's association with type 2 diabetes [21]; [22]. With regard to the recent identification of melatonin receptors in pancreatic β-cells [34]; [35] and melatonin's regulatory (i.e., inhibitory) effect on insulin secretion of INS1 insulinoma cells and rat pancreatic islets in vitro [36]–[38], it is conceivable that common genetic variation within *MTNR1B* affects β-cell function directly.

Interestingly, the minor alleles of the *MTNR1B* SNPs rs10830962, rs4753426, and rs10830963 were associated with reduced insulin secretion, whereas the minor allele of rs3781638 was associated with higher insulin release during the OGTT, and this opposite effect was accordingly reflected at the level of fasting plasma glucose concentrations. Since none of the common informative HapMap SNPs is located within the coding region of the *MTNR1B* gene, common genetic variation within this gene presumably affects *MTNR1B* expression by altering transcription factor binding sites. As gene expression is regulated in a very complex way, frequently involving many transcription factors simultaneously, it does not appear far-fetched to assume that SNPs within different cis-acting DNA elements can alter gene expression in opposite directions.

In addition to its insulin secretion-modulating effect, SNP rs3781638 also affected insulin sensitivity, as assessed by insulin and glucose data in the fasting state and during OGTT, and this effect was most obvious in states known to be associated with insulin resistance (impaired fasting glycemia and impaired glucose tolerance). An association with clamp-derived insulin sensitivity was not observed. This discrepancy could be due to the limited power of the clamped subgroup. Alternatively, this SNP could affect hepatic insulin sensitivity which is better reflected by the former parameters, whereas clamp-derived insulin sensitivity predominantly represents skeletal muscle insulin sensitivity. Our finding that SNP rs3781638 was associated with measures of hepatic insulin clearance not only in the overall cohort but also in the clamped subgroup argues against a power problem in the clamped subgroup and favours a role of this SNP in hepatic insulin sensitivity. To ultimately clarify this issue, further studies with better, e.g., tracer-based, methods are required.

In conclusion, common genetic variation within *MTNR1B* determines glucose-stimulated insulin secretion and plasma glucose concentrations. The marked impact on β-cell function might represent the prevailing pathomechanism how genetic variation within this gene increases the type 2 diabetes risk.

## Supporting Information

Table S1 Data represent means±SD. For statistical analysis, data were log-transformed and adjusted. BMI, body fat, and waist circumference were adjusted for gender and age. Plasma glucose levels, indices of insulin sensitivity, and the disposition index were adjusted for gender, age, and BMI. Other indices of insulin secretion were adjusted for gender, age, BMI, and ISI (OGTT). p1-p-value after adjustment as described; p2-p-value after additional adjustment for family history of diabetes. Significance levels withstanding Bonferroni correction for multiple comparisons are marked in bold letters. AUC-area under the curve; HOMA-IR-homeostasis model assessment of insulin resistance; ISI-insulin sensitivity index; SNP-single nucleotide polymorphism. *subgroup (N = 394).
(0.08 MB DOC)

Table S2 Data represent means±SD. For statistical analysis, data were log-transformed and adjusted. BMI, body fat, and waist circumference were adjusted for gender and age. Plasma glucose levels, indices of insulin sensitivity, and the disposition index were adjusted for gender, age, and BMI. Other indices of insulin secretion were adjusted for gender, age, BMI, and ISI (OGTT). p1-p-value after adjustment as described; p2-p-value after additional adjustment for family history of diabetes. Significance levels withstanding Bonferroni correction for multiple comparisons are marked in bold letters. AUC-area under the curve; HOMA-IR-homeostasis model assessment of insulin resistance; ISI-insulin sensitivity index; SNP-single nucleotide polymorphism. *subgroup (N = 394).
(0.06 MB DOC)

Table S3 Data represent means±SD. For statistical analysis, data were log-transformed and adjusted. BMI, body fat, and waist circumference were adjusted for gender and age. Plasma glucose levels, indices of insulin sensitivity, and the disposition index were adjusted for gender, age, and BMI. Other indices of insulin secretion were adjusted for gender, age, BMI, and ISI (OGTT). p1-p-value after adjustment as described; p2-p-value after additional adjustment for family history of diabetes. Significance levels withstanding Bonferroni correction for multiple comparisons are marked in bold letters. AUC-area under the curve; HOMA-IR-homeostasis model assessment of insulin resistance; ISI-insulin sensitivity index; SNP-single nucleotide polymorphism. *subgroup (N = 119).
(0.08 MB DOC)

Table S4 Data represent means±SD. For statistical analysis, data were log-transformed and adjusted. BMI, body fat, and waist circumference were adjusted for gender and age. Plasma glucose levels, indices of insulin sensitivity, and the disposition index were adjusted for gender, age, and BMI. Other indices of insulin secretion were adjusted for gender, age, BMI, and ISI (OGTT). p1-p-value after adjustment as described; p2-p-value after additional adjustment for family history of diabetes. Significance levels withstanding Bonferroni correction for multiple comparisons are marked in bold letters. AUC-area under the curve; HOMA-IR-homeostasis model assessment of insulin resistance; ISI-insulin sensitivity index; SNP-single nucleotide polymorphism. *subgroup (N = 119).
(0.06 MB DOC)
