# Supplementary material for: Polymorphisms within the Novel Type 2 Diabetes Risk Locus MTNR1B Determine β-Cell Function
Source: PLoS One. 2008 Dec 17;3(12):e3962. doi: 10.1371/journal.pone.0003962 (PMC2597741; doi:10.1371/journal.pone.0003962)
Supplement: Table S1 — Data represent means±SD. For statistical analysis, data were log-transformed and adjusted. BMI, body fat, and waist circumference were adjusted for gender and age. Plasma glucose levels, indices of insulin sensitivity, and the disposition index were adjusted for gender, age, and BMI. Other indices of insulin secretion were adjusted for gender, age, BMI, and ISI (OGTT). p1-p-value after adjustment as described; p2-p-value after additional adjustment for family history of diabetes. Significance levels withstanding Bonferroni correction for multiple comparisons are marked in bold letters. AUC-area under the curve; HOMA-IR-homeostasis model assessment of insulin resistance; ISI-insulin sensitivity index; SNP-single nucleotide polymorphism. *subgroup (N = 394). (0.08 MB DOC) [file pone.0003962.s001.doc]

**Supplementary Table S1.** Associations of *MTNR1B* SNPs rs10830962, rs4753426, and rs12804291 with anthropometrics, insulin sensitivity, and insulin secretion in normal glucose-tolerant subjects (N=1139).

| SNP | rs10830962 | | |  |  | rs4753426 | | |  |  | rs12804291 | | |  |  |
| --- | --- | --- | --- | --- | --- | --- | --- | --- | --- | --- | --- | --- | --- | --- | --- |
| Genotype | CC | CG | GG | p_1_ | p_2_ | TT | TC | CC | p_1_ | p_2_ | CC | CT | TT | p_1_ | p_2_ |
| N | 409 | 556 | 173 | - | - | 302 | 578 | 259 | - | - | 905 | 219 | 15 | - | - |
| Age (y) | 38 ±13 | 37 ±13 | 37 ±12 | - | - | 38 ±12 | 37 ±13 | 37 ±12 | - | - | 37 ±13 | 37 ±12 | 39 ±13 | - | - |
| BMI (kg/m²) | 27.9 ±6.9 | 27.1 ±7.0 | 27.7 ±7.2 | 0.1 | 0.09 | 28.1 ±6.7 | 27.0 ±6.8 | 27.9 ±7.6 | 0.0332 | 0.0120 | 27.4 ±7.0 | 27.4 ±6.9 | 29.9 ±9.2 | 0.5 | 0.5 |
| Body fat (%) | 30 ±11 | 29 ±10 | 30 ±11 | 0.0372 | 0.0303 | 31 ±10 | 29 ±10 | 30 ±11 | 0.0048 | 0.0034 | 30 ±10 | 29 ±11 | 35 ±10 | 0.2 | 0.2 |
| Waist circum-ference (cm) | 92 ±16 | 90 ±16 | 91 ±15 | 0.2 | 0.2 | 92 ±16 | 90 ±15 | 92 ±16 | 0.0361 | 0.0178 | 91 ±15 | 91 ±16 | 88 ±13 | 0.6 | 0.7 |
| Fasting glucose (mM) | 4.85 ±0.39 | 4.92 ±0.40 | 4.93 ±0.42 | **0.0009** | **0.0012** | 4.87 ±0.41 | 4.88 ±0.39 | 4.96 ±0.39 | 0.0049 | 0.0052 | 4.90 ±0.40 | 4.89 ±0.40 | 4.99 ±0.42 | 0.9 | 0.8 |
| Glucose 120min OGTT (mM) | 5.54 ±1.16 | 5.66 ±1.13 | 5.68 ±1.10 | 0.0187 | 0.0309 | 5.60 ±1.15 | 5.61 ±1.14 | 5.65 ±1.11 | 0.3 | 0.3 | 5.61 ±1.14 | 5.62 ±1.10 | 6.10 ±1.28 | 0.6 | 0.7 |
| HOMA-IR (U) | 2.04 ±1.54 | 1.98 ±1.68 | 2.07 ±1.89 | 0.8 | 0.8 | 2.10 ±1.64 | 1.94 ±1.60 | 2.09 ±1.82 | 1.0 | 1.0 | 2.00 ±1.57 | 2.06 ±2.04 | 2.43 ±1.23 | 0.5 | 0.5 |
| ISI, OGTT (U) | 18.5 ±11.4 | 18.8 ±10.7 | 19.2 ±11.7 | 0.9 | 0.8 | 18.4 ±11.2 | 19.1 ±11.1 | 18.4 ±11.2 | 0.8 | 0.7 | 18.8 ±11.2 | 19.0 ±11.2 | 14.6 ±7.9 | 0.8 | 0.8 |
| ISI, clamp (U)* | 0.096 ±0.068 | 0.093 ±0.049 | 0.093 ±0.053 | 0.7 | 0.7 | 0.092 ±0.061 | 0.095 ±0.056 | 0.095 ±0.049 | 0.6 | 0.5 | 0.095 ±0.058 | 0.093 ±0.050 | 0.058 ±0.025 | 0.2 | 0.2 |
| 1^st^-phase insulin secretion (nM) | 1.34 ±0.86 | 1.21 ±0.75 | 1.23 ±0.84 | **<0.0001** | **0.0001** | 1.34 ±0.88 | 1.23 ±0.76 | 1.23 ±0.81 | 0.0043 | 0.0077 | 1.26 ±0.82 | 1.24 ±0.75 | 1.30 ±0.54 | 0.6 | 0.5 |
| Insulinogenic index (·10^-9^) | 182 ±290 | 138 ±444 | 139 ±123 | **<0.0001** | **<0.0001** | 133 ±574 | 170 ±275 | 142 ±133 | 0.0315 | 0.0338 | 167 ±235 | 101 ±665 | 145 ±77 | 0.2 | 0.2 |
| AUC C-pep/AUC glc (·10^-9^) | 337 ±112 | 322 ±103 | 315 ±99 | 0.0405 | 0.05 | 335 ±113 | 327 ±104 | 314 ±103 | 0.0082 | 0.0148 | 327 ±106 | 323 ±109 | 315 ±109 | 0.5 | 0.5 |
| Disposition index (U) | 19.5 ±8.9 | 18.2 ±8.1 | 18.2 ±8.8 | **0.0005** | **0.0008** | 19.2 ±8.9 | 18.8 ±8.4 | 17.8 ±8.4 | 0.0075 | 0.0098 | 18.7 ±8.6 | 18.6 ±8.4 | 15.9 ±4.7 | 0.7 | 0.6 |

Data represent means ±SD. For statistical analysis, data were log-transformed and adjusted. BMI, body fat, and waist circumference were adjusted for gender and age. Plasma glucose levels, indices of insulin sensitivity, and the disposition index were adjusted for gender, age, and BMI. Other indices of insulin secretion were adjusted for gender, age, BMI, and ISI (OGTT). p_1_ – p-value after adjustment as described; p_2_ – p-value after additional adjustment for family history of diabetes. Significance levels withstanding Bonferroni correction for multiple comparisons are marked in bold letters. AUC – area under the curve; HOMA-IR – homeostasis model assessment of insulin resistance; ISI – insulin sensitivity index; SNP – single nucleotide polymorphism. *subgroup (N=394).
